# Usefulness of the skeletal muscle index in postoperative ileus of colorectal cancer patients: a retrospective cohort study

**DOI:** 10.1186/s12893-022-01887-3

**Published:** 2022-12-31

**Authors:** Maho Sasaki, Tatsunari Fukuoka, Masatsune Shibutani, Atsushi Sugimoto, Kiyoshi Maeda, Masaichi Ohira

**Affiliations:** 1 Department of Gastroenterological Surgery, Osaka Metropolitan University Graduate School of Medicine, 1-4-3 Asahimachi, Abeno-Ku, Osaka, 545-8585 Japan; 2 Department of Gastroenterological Surgery, Baba Memorial Hospital, 4-244, Hamaderafunaochohigashi, Nishi-Ku, Sakai, Osaka 592-8341 Japan

**Keywords:** Colorectal cancer, Postoperative ileus, Skeletal muscle index

## Abstract

**Background:**

Postoperative complications of colorectal cancer mainly include infections (surgical site infection, remote infection, etc.), post-operative ileus (POI), and anastomosis leakage. POI reportedly prolongs the hospital stay and increases medical costs. Therefore, predicting POI is very important. There have been some reports on the relationships between sarcopenia and postoperative complications in colorectal cancer patients, but none have been limited to POI. We therefore conducted a retrospective clinical study with a logistic regression analysis to confirm the risk factors for ileus after colorectal cancer surgery.

**Methods:**

We retrospectively analyzed 213 patients who underwent elective oncological colorectal surgery for colorectal cancer from November 2017 to July 2021. The skeletal muscle mass (SMM, kg) was estimated using a whole-body bioelectrical impedance analysis (BIA), and the skeletal muscle index (SMI) was calculated as the SMM/height^2^. We divided all patients into 2 groups based on a low SMI (male < 8.958 kg/m^2^, female < 8.443 kg/m^2^) or normal SMI. Preoperative and intraoperative factors as well as, postoperative outcomes were then compared between the two groups.

**Results:**

The median (range) age of the 213 included patients was 72.0 (33–91) years old. Complications were noted in 96 patients (45.1%), including 21 (9.9%) with POI. There were 68 (31.9%) low SMI patients. POI occurred significantly more frequently in low SMI patients (19.1%) than in normal SMI patients (5.5%) (p = 0.005). In the multivariate analysis, bleeding (p = 0.039) and a low SMI (p = 0.031) were significantly associated with POI. In addition, a propensity score matching analysis was performed to further reduce the selection bias. As a result, a low SMI was the only independent POI predictor among the 78 matched cases.

**Conclusion:**

A preoperative low SMI in colorectal cancer patients was considered a risk factor for POI.

## Introduction

Colorectal cancer (CRC) has the third-highest incidence and second-highest mortality rate among cancers, with over 1.93 million cases and 916,000 deaths a year [1]. Surgery is the only curative option. However, postoperative complications occur in a substantial number of cases [2, 3].

The definition of POI includes symptoms such as nausea, vomiting, and abdominal bloating as well as dilation of the small intestine without obvious obstruction as confirmed on imaging [4]. Post-operative ileus (POI) increases post-operative morbidity and prolongs the hospital stay while increasing the medical costs [5–8]. Postoperative complications, including POI, after surgery for CRC are reportedly associated with delays in adjuvant chemotherapy, and patients who receive delayed adjuvant chemotherapy have higher recurrence rates and a worse overall survival than those who receive chemotherapy within 8 weeks of surgical treatment [9]. Among the postoperative complications of CRC, the incidence of POI is reported to be 10%-30% after abdominal surgery and 15%–30% after colon [4, 8] and rectal surgery [10–12]. Therefore, predicting POI is very important in CRC surgery.

Furthermore, preoperative sarcopenia has been attracting attention in recent years as a predictor of postoperative complications. Sarcopenia was initially described in 1989 as a condition in which muscle mass decreases with age [13]. Sarcopenia is a condition with many causes and varying outcomes. While sarcopenia is mainly observed in older people, it can also develop in younger adults, as is likewise the case for dementia and osteoporosis. Sarcopenia is divided into primary sarcopenia in a narrow sense and secondary sarcopenia in a broad sense, depending on its origin. Primary sarcopenia is a decrease in muscle mass with aging, while secondary sarcopenia is a decrease in muscle mass associated with decreased activity, malnutrition, and diseases, such as organ failure and malignant tumors [14, 15].

Among CRC patients, the number of patients with primary sarcopenia due to aging of patients and secondary sarcopenia due to malnutrition or cancer-bearing status is increasing. Therefore, it is very important to understand the preoperative condition of the patient. There have been some reports on the relationships between sarcopenia and postoperative complications in CRC patients [16–18], but there have been no studies limited to POI thus far. We therefore investigated the relationship between sarcopenia and POI in CRC patients.

## Materials and methods

### Patients and specimens

We retrospectively analyzed 213 consecutive patients with CRC who underwent elective oncological colorectal surgery at Osaka City University Hospital from November 2017 to July 2021. We excluded patients with double cancer and those who received preoperative chemo-radiotherapy, neoadjuvant chemotherapy and emergency surgery. The following clinical and surgical data were collected from electronic medical records: age, gender, body mass index (BMI), patient history, and blood test results.

Patients’ clinicopathological characteristics are summarized in Table 1. The histological diagnosis was based on the World Health Organization criteria [19]. Pathologic staging was performed according to the 8th edition of the Union for International Cancer Control TNM classification of malignant tumors [20].

**Table 1:** Clinicopathologic characteristics of 213 colorectal cancer patients

Clinicopathologic features	n = 213
*Sex*	
Male	126 (59.2%)
Female	87 (40.8%)
Age (years)*	
Mean (SD)	72 (11.3)
BMI*	
Mean (SD)	23.2 (4.1)
*Comorbidities*	
Cardiac disease	37 (17.3%)
Lung disease	23 (10.8%)
Diabetes mellitus	46 (21.6%)
*Location of tumor*	
C	17
A	42
T	20
D	8
S	46
R	80
*pT stage*	
Tis-T2	93 (43.7%)
T3-T4	120 (56.3%)
*pN stage*	
Positive	60 (28.2%)
Negative	153 (61.8%)
*pStage*	
0	10
I	73
II	63
III	49
IV	18
*Surgical procedure*	
Laparoscopy	187 (87.8%)
Open	26 (12.2%)
Operative time (min)*	
Mean (SD)	265.9 (111.9)
Intraoperative bleeding (ml)*	
Mean (SD)	117.5 (332.1)
*Postoperative complications*	
All	96 (45.1%)
Surgical site infection	50 (23.5%)
Anastomotic leakage	17 (8.0%)
Remote infection	12 (5.6%)
POI	21 (9.9%)
CDC grade	
I	21 (9.9%)
II	53 (24.9)
IIIa	20 (9.4%)
IIIb	6 (2.8%)
IVa	1 (0.5%)

Continuous parameters are presented as mean (SD)*, and categorical parameters are presented as n (%), *C* cecum cancer, *A* ascending colon cancer, *T* transverse colon cancer, *D* descending colon cancer, *S* sigmoid colon cancer, *R* rectum cancer, *Tis* carcinoma in situ, *POI* postoperative ileus, *CDC* Clavien-Dindo classification, *SD* standard deviation

### Postoperative complications

The results of postoperative complications are also shown in Table 1. Postoperative complications were defined as in-hospital morbidity or mortality occurring within 30 days of surgery and classified according to the Clavien-Dindo Classification (CDC) [21]. Surgical site infections (SSIs), anastomotic leakage, remote infection, and postoperative ileus (POI) were included among postoperative complications.

### POI

POI was defined in cases with symptoms such as nausea, vomiting, and abdominal bloating as well as dilation of the small intestine without obvious obstruction confirmed on imaging [4].

### Skeletal muscle index (SMI)

**Fig. 1:**
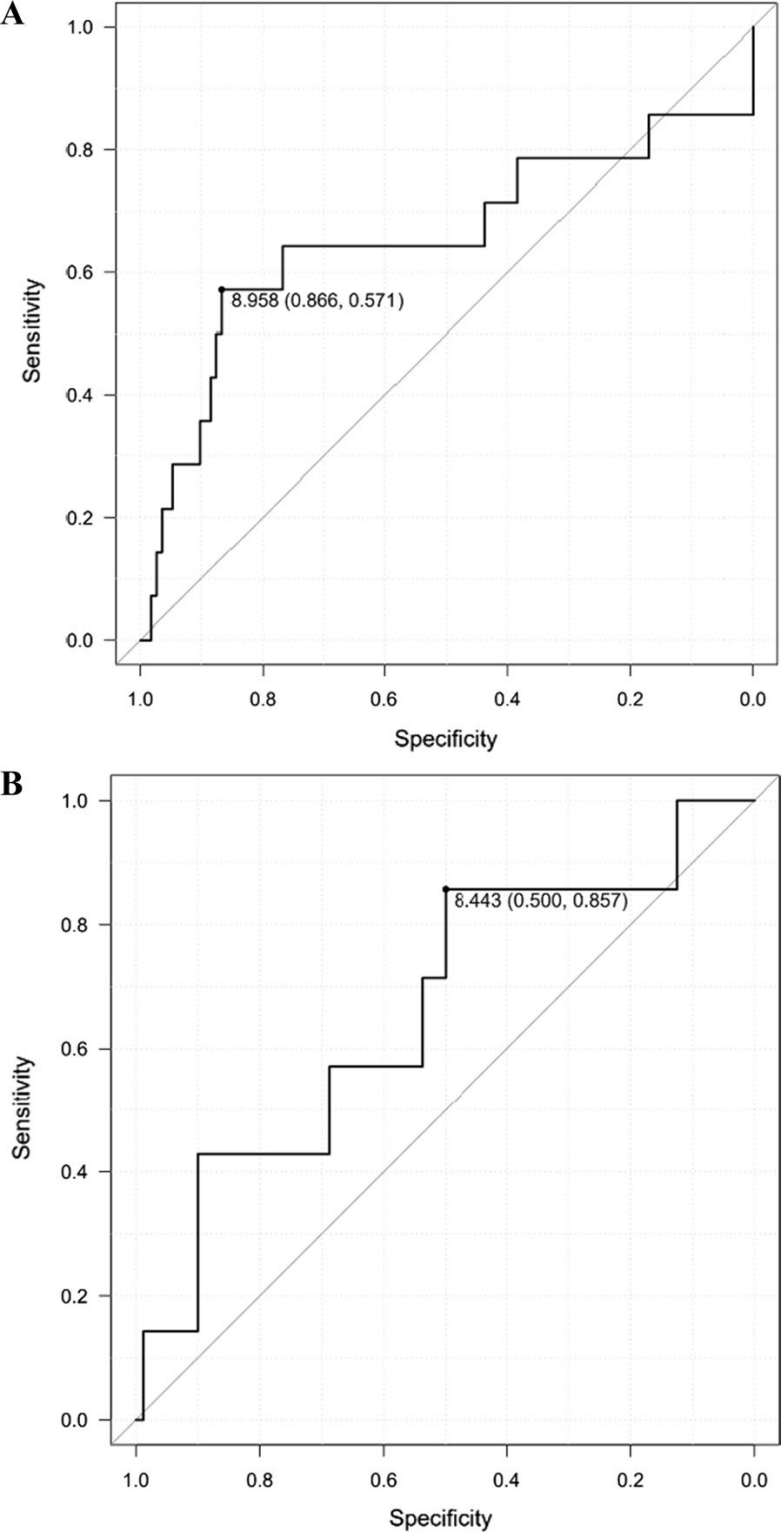
**A** ROC curve for Male. The cut-off values was 8.958 for male by the point on the ROC curve predicting POI. *POI* postoperative ileus. **B** ROC curve for Female. The cut-off values was 8.443 for female by the point on the ROC curve predicting POI. *POI* postoperative ileus

All patients had their skeletal muscle mass (SMM) measured by a bioelectrical impedance analysis (BIA) method using an InBody®S10 (InBody Japan, Tokyo, Japan) two days before their surgery. Using the formula for calculating the SMI (kg/m^2^) [SMM/(patient height)^2^] we generated SMIs for each patient. The low SMI group (L group) was defined as those with values lower than the sex-specific cut-off values (8.958 and 8.443 kg/m^2^ for males and females, respectively) by the point on the receiver operating characteristic (ROC) curve predicting POI (Fig. 1A, B). All patients were classified into the L group or normal SMI group (N group) based on these cut-off values.

### Propensity score matching (PSM)

A PSM analysis was used to adjust for differences in clinicopathological characteristics between the L and N groups. The variables were sex, age (continuous), comorbidities (pulmonary disease and diabetes mellitus), BMI (continuous), Onodera’s prognostic nutritional index (PNI = 10 × Alb (g/dl) + 0.005 × lymphocytes [/μl], continuous), all of which are associated with the SMI. Each variable was multiplied by a coefficient that was calculated using a logistic regression analysis, and the sum of these values was taken as the propensity score for individual patients. The L and N groups were matched 1:1 based on their propensity scores using nearest-neighbor matching without replacement (caliper size: 0.2). If no suitable match within the 0.2 caliper size remained in the control group, the exposed patient was excluded from the matched data set.

### Statistical analyses

The data of continuous variables were described as the median (interquartile range [IQR]). A univariate analysis was performed by the chi-squared test or Fisher's exact for categorical variables. To identify predictors of POI, multivariate analyses including variables with *p* < 0.1 in the univariate analyses were performed using the logistic regression model. Odds ratios (ORs) and 95% confidence intervals (CIs) were calculated. All statistical analyses were performed with EZR (Saitama Medical Center, Jichi Medical University, Saitama, Japan), which is a graphical user interface for R (The R Foundation for Statistical Computing, Vienna, Austria). More precisely, it is a modified version of R commander designed to add statistical functions frequently used in biostatistics [22].

## Results

### Patient characteristics

A total of 213 (126 male and 87 female) patients were identified in this study. The median age was 72.0 (33–91) (Table 1). According to the ROC curve analysis, all patients were classified into two groups: L group (n = 68, male SMI < 8.958 kg/m^2^, female SMI < 8.443 kg/m^2^) and the N group (n = 145) based on the SMI cut-off values (Fig. 1A, B). Thirty-seven patients had comorbid cardiac disease, 23 had lung disease (defined as a % vital capacity of < 60% or a % forced expiratory volume in 1.0 s of < 50%), and 46 had diabetes mellitus (DM).

**Fig. 2:**
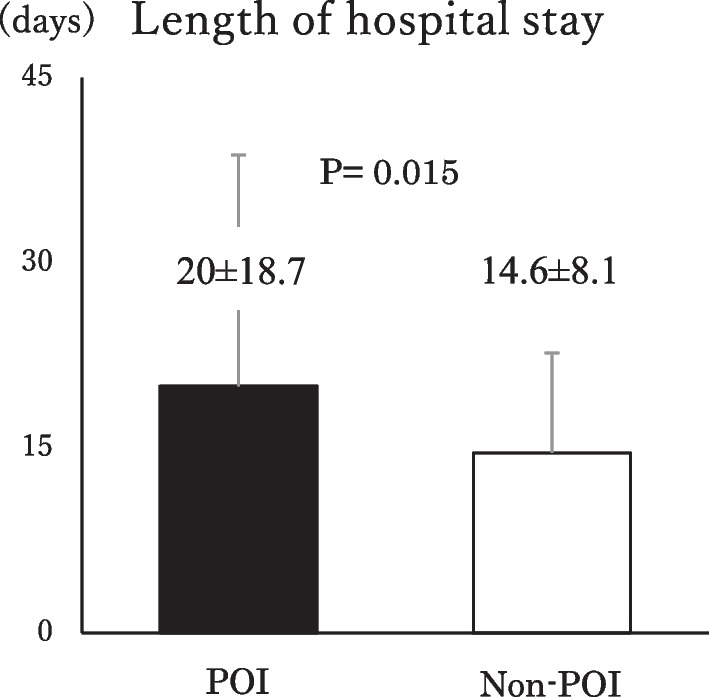
Length of hospital stay. ■: POI, □: Non-POI. Patients with POI had significantly longer postoperative hospital stays (mean ± SD = 20 ± 18.7) than non-onset patients (mean ± SD = 14.6 ± 8.1, P = 0.015). *POI* postoperative ileus

Postoperative complications were observed in 96 cases (45.1%). SSI was observed in 50 cases, anastomosis leakage was observed in 17 cases, remote infection was observed in 12 cases and POI was observed in 21 cases (9.9%). The POI onset occurred on average 3.2 days after surgery, and 2 of 21 cases required ileus tube insertion, while 7 cases required nasogastric (NG) tube insertion. It took an average of 5.6 days for the condition to improve. Patients with POI had a significantly longer postoperative hospital stay than the non-onset patients (Fig. 2).

### Correlation between the SMI and clinicopathological characteristic factors

Regarding preoperative factors, the L group was had a significantly older age, higher rate of female participants, lower preoperative BMI, higher rate of lung diseases and non-diabetes mellitus than the N group, according to a univariate analysis (Table 2). No significant differences were found between the groups in any of the cancer progression or intraoperative factors. Regarding postoperative complication factors, the frequency of POI was significantly higher in the L group than in the N group (p = 0.005).

**Table 2:** Association between preoperative decrease in skeletal muscle mass and clinical pathological background

Back ground	Low SMI (n = 68)	Normal SMI (n = 145)	p-value
*Sex*			
Female	45 (66.2%)	42 (29.0%)	< 0.001
Age (years)*			
Mean (SD)	73.1 (11.2)	68.4 (11.0)	0.004
BMI*			
Mean (SD)	20.6 (3.3)	24.4 (3.8)	< 0.001
PNI*			
Mean (SD)	45.3 (6.3)	47.0 (6.5)	0.076
*GPS*			
2 over	4 (5.9%)	13 (9.0%)	0.591
Alb (g/dL)*			
Mean (SD)	3.79 (0.5)	3.88 (0.5)	0.268
*Cardiac disease*			
Positive	9 (13.2%)	28 (19.3%)	0.334
*Lung disease*			
Positive	12 (17.6%)	11 (7.6%)	0.034
*Diabetes mellitus*			
Positive	7 (10.3%)	39 (26.9%)	0.007
*Tumor location*			
Rectal tumor	21 (30.9%)	59 (40.7%)	0.176
*Tumor size*			
≧3.0 cm	40 (61.5%)	83 (58.9%)	0.761
*pT stage*			
T3 over	40 (58.8%)	80 (55.2%)	0.658
*pN stage*			
Positive	23 (33.8%)	37 (25.5%)	0.253
*P*			
Positive	4 (5.9%)	4 (2.8)	0.270
*H*			
Positive	4 (5.9%)	8 (5.5%)	1.0
*Surgical procedure*			
Laparoscopy	57 (83.8%)	130 (89.7%)	0.263
*Stoma*			
Positive	8 (11.8%)	23 (15.9%)	0.534
*Operative time (min)*			
> 240	34 (50.0%)	76 (52.4%)	0.770
*Bleeding (min)*			
≧101	12 (17.6%)	28 (19.3%)	0.852
*CDC*			
Grade 3 over	5 (7.4%)	22 (15.2%)	0.126
*Complications*			
SSI	11 (16.2%)	39 (26.9%)	0.118
Anastomotic leakage	3 (4.4%)	14 (9.7%)	0.279
Remote infection	5 (7.4%)	7 (4.8%)	0.527
POI	13 (19.1%)	8 (5.5%)	0.005

Continuous parameters are presented as mean (SD)*, and categorical parameters are presented as n (%), *SMI* skeletal muscle index, *BMI* body mass index, *PNI* prognostic nutritional index, *GPS* glasgow prognostic score, *Alb* albumin*, Lung disease* was defined as a condition with % vital capacity < 60% or a % force expiratory volume in 1.0 s < 50%, *TNM* tumor node metastasis, *P* peritoneal dissemination, *H* hepatic metastasis, *CDC* Clavien–Dindo classification, *SSI* surgical site infection, *POI* post operative ileus, *P* < 0.05

### Univariate and multivariate analyses for POI

Table 3 shows the relationship of the clinicopathological characteristics with POI. A low BMI (p = 0.015), Stoma (p = 0.014), bleeding (p = 0.005), and low SMI (p = 0.003) were significantly associated with POI in the univariate analysis. The multivariate analysis including variables with p < 0.1 in univariate analyses, revealing that bleeding (p = 0.039, OR = 3.850, 95% CI 1.07–13.80) and a low SMI (p = 0.031, OR = 3.490, 95% CI 1.12–10.90) were independent predictors of POI.

**Table 3:** Association between POI and clinicopathological characteristics

Variable	Univariate analysis			Multivariate analysis		
	Hazard	95%CI	P-value	Hazard	95%CI	P-value
*Sex*						
Female	0.70	0.27–1.81	0.463			
Age						
	1.000	0.96–1.04	0.981			
BMI						
	0.833	0.719–0.965	**0.015**	0.901	0.770–1.05	0.192
PNI						
	0.959	0.896–1.03	0.235			
GPS						
2 over	0.550	0.069–4.37	0.572			
Alb						
	0.795	0.343–1.84	0.594			
*Cardiac disease*						
Positive	1.13	0.358–3.59	0.831			
*Lung disease*						
Positive	1.43	0.388–5.30	0.589			
*Diabetes mellitus*						
Positive	1.15	0.398–3.33	0.795			
*Tumor location*						
Rectal tumor	1.03	0.406–2.59	0.957			
*Tumor size*						
≧3.0 cm	0.889	0.357–2.21	0.800			
*pT stage*						
T3 over	1.04	0.417–2.58	0.938			
*pN stage*						
Positive	0.778	0.272–2.23	0.641			
*P*						
Positive	1.320	0.155–11.30	0.799			
*H*						
Positive	0.823	0.101–6.71	0.855			
*Surgical procedure*						
Laparoscopy	2.54	0.846–7.66	**0.097**	0.849	0.206–3.50	0.820
*Stoma*						
Positive	3.50	1.28–9.54	**0.014**	2.710	0.83–8.84	0.099
*Operative time (min)*						
> 240	1.2	0.48–2.95	0.698			
*Bleeding (ml)*						
≧101	3.90	1.51–10.00	**0.005**	3.850	1.07–13.80	**0.039**
*SMI*						
Low SMI	4.05	1.59–10.30	**0.003**	3.490	1.12–10.90	**0.031**
*CDC*						
Grade 3 over	1.17	0.32–4.26	0.816			
*Complications*						
SSI	0.769	0.246–2.40	0.651			
Anastomotic leakage	0.550	0.069–4.37	0.572			
Remote infection	0.823	0.101–6.71	0.855			

Bold text indicates a statistically significant difference with a *P*-value < 0.05

*CI* confidence interval, *P* < 0.05

### PSM

To reduce the possibility of selection bias, PSM was performed. After PSM, 39 matched pairs were created. The patient characteristics before and after PSM are shown in Table 4. After PSM, the two groups did not show any significant differences in clinicopathological characteristics.

**Table 4:** Clinicopathological characteristics before and after propensity score matching

Variable	All patients			Matched patients		
	Low SMI (n = 68)	Normal (n = 145)	P-value	Low SMI (n = 39)	Normal (n = 39)	P-value
*Sex*						
Male	23 (33.8%)	103 (71.0%)		21 (53.8%)	17 (43.6%)	
Female	45 (66.2%)	42 (29.0%)	** < 0.001**	18 (46.2%)	22 (56.4%)	0.497
Age (years)*						
Mean (SD)	73.1 (11.2)	68.4 (11.0)	**0.004**	72.9 (12.3)	70.7 (10.9)	0.401
BMI*						
Mean (SD)	20.6 (3.3)	24.4 (3.8)	** < 0.001**	21.5 (3.67)	21.7 (2.32)	0.825
PNI*						
Mean (SD)	45.3 (6.3)	47.0 (6.5)	0.076	45.7 (6.94)	46.7 (5.82)	0.512
*GPS*						
0.1	64 (94.1%)	132 (91.0%)		36 (92.3%)	35 (89.7%)	
2	4 (5.9%)	13 (9.0%)	0.591	3 (7.7%)	4 (10.3%)	1.000
Alb (g/dL)*						
Mean (SD)	3.79 (0.5)	3.88 (0.5)	0.268	3.81 (0.5)	3.87 (0.5)	0.643
*Cardiac disease*						
Positive	9 (13.2%)	28 (19.3%)				
Negative	59 (86.8%)	117 (80.7%)	0.334			
*Lung disease*						
Positive	12 (17.6%)	11 (7.6%)		8 (20.5%)	6 (15.4%)	
Negative	56 (82.4%)	134 (92.4%)	**0.034**	31 (79.5%)	33 (84.6%)	0.769
*Diabetes mellitus*						
Positive	7 (10.3%)	39 (26.9%)		6 (15.4%)	7 (17.9%)	
Negative	61 (89.7%)	106 (73.1%)	**0.007**	33 (84.6%)	32 (82.1%)	1.000
*Tumor location*						
Rectum	21 (30.9%)	59 (40.7%)		13 (33.3%)	16 (41.0%)	
Others	47 (69.1%)	86 (59.3%)	0.176	26 (66.7%)	23 (59.0%)	0.640
*Tumor size (cm)*						
< 3.0	25 (38.5%)	58 (41.1%)		24 (63.2%)	21 (55.3%)	
≧3.0	40 (61.5%)	83 (58.9%)	0.761	14 (36.8%)	17 (44.7%)	0.641
*pT stage*						
≧3	40 (58.8%)	80 (55.2%)		24 (61.5%)	21 (53.8%)	
0–2	28 (41.2%)	65 (44.8%)	0.658	15 (38.5%)	18 (46.2%)	0.647
*pN stage*						
Positive	23 (33.8%)	37 (25.5%)		12 (30.8%)	10 (25.6%)	
Negative	45 (66.2%)	108 (74.5%)	0.253	27 (69.2%)	29 (74.4%)	0.802
*P*						
Positive	4 (5.9%)	4 (2.8%)		2 (5.1%)	2 (5.1%)	
Negative	64 (94.1%)	141 (97.2%)	0.270	37 (94.9%)	37 (94.9%)	1.000
*H*						
Positive	4 (5.9%)	8 (5.5%)		2 (5.1%)	3 (7.7%)	
Negative	64 (94.1%)	137 (94.5%)	1.000	37 (94.9%)	36 (92.3%)	1.000
*Surgical procedure*						
Open	11 (16.2%)	15 (10.3%)		6 (15.4%)	4 (10.3%)	
Lap	57 (83.8%)	130 (89.7%)	0.263	33 (84.6%)	35 (89.7%)	0.737
*Stoma*						
Positive	8 (11.8%)	23 (15.9%)		6 (15.4%)	4 (10.3%)	
Negative	60 (88.2%)	122 (84.1%)	0.534	33 (84.6%)	35 (89.7%)	0.737
*Operative Time (min)*						
> 240	34 (50.0%)	69 (47.6%)		20 (51.3%)	16 (41.0%)	
≦240	34 (50.0%)	76 (52.4%)	0.770	19 (48.7%)	23 (59.0%)	1.000
*Bleeding (ml)*						
≧101	12 (17.6%)	28 (19.3%)		6 (15.4%)	6 (15.4%)	
< 101	56 (82.4%)	117 (80.7%)	0.852	33 (84.6%)	33 (84.6%)	0.496

Bold text indicates a statistically significant difference with a *P*-value < 0.05

Continuous parameters are presented as mean (SD)*, and categorical parameters are presented as n (%), *P* < 0.05

### Univariate and multivariate analyses for POI in 78 matched patients

Table 5 shows the results of the univariate analysis for POI in 78 matched patients. The univariate analysis showed Sex (p = 0.081) and low BMI (p = 0.085) tended to increase POI. And low SMI (p = 0.025) was associated with POI in a univariate analysis. The multivariate analysis including variables with p < 0.1 in univariate analyses, revealing that a low SMI (p = 0.031, OR = 10.80, 95% CI 1.25–93.20) was the only independent predictor of POI in 78 matched patients.

**Table 5:** Univariate and multivariate analyses for POI risk factors in matched patients

Back Ground	POI (n = 10)	Non-POI (n = 68)	Univariate analysis	Multivariate analysis		
			P-value	Hazard	95%CI	P-value
*Sex*						
Female	2 (20.0%)	35 (51.5%)	**0.081**	0.309	0.051–1.87	0.201
Age (years)*						
mean (SD)	70.30 (18.20)	72.57 (10.15)	0.553			
BMI*						
Mean (SD)	20.04 (1.20)	21.72 (3.00)	**0.085**	0.877	0.649–1.19	0.394
PNI*						
Mean (SD)	45.67 (4.82)	46.16 (6.93)	0.829			
*GPS*						
2 over	0 (0.0%)	7 (10.3%)	0.992			
Alb (g/dl)*						
mean (SD)	3.85 (0.27)	3.83 (0.54)	0.914			
*Lung Disease*						
Positive	1 (10.0%)	13 (19.1%)	0.492			
*Diabetes mellitus*						
Positive	1 (10.0%)	12 (17.6%)	0.551			
*Tumor location*						
Rectal tumor	3 (30.0%)	23 (33.8%)	0.811			
*Tumor Size*						
≧3.0 cm	7 (70.0%)	36 (54.5%)	0.364			
*pT stage*						
T3 over	7 (70.0%)	37 (54.4%)	0.360			
*pN stage*						
Positive	3 (30.0%)	20 (29.4%)	0.970			
*P*						
Positive	1 (10.0%)	2 (2.9%)	0.308			
*H*						
Positive	0 (0.0%)	5 (7.4%)	0.993			
*Surgical* *procedure*						
Laparoscopy	8 (80.0%)	58 (85.3%)	0.666			
*Stoma*						
Positive	2 (20.0%)	7 (10.3%)	0.379			
*Operative time (min)*						
> 240	5 (50.0%)	30 (44.1%)	0.727			
*Bleeding (ml)*						
≧101	3 (30.0%)	13 (19.1%)	0.431			
*SMI*						
Low SMI	9 (90%)	30 (44.1%)	**0.025**	10.80	1.25–93.20	**0.031**
*CDC*						
Grade 3 over	0 (0.0%)	11 (16.2%)	0.993			
*Complications*						
SSI	3 (30.0%)	16 (23.5%)	0.657			
Anastomotic leakage	0 (0.0%)	9 (13.2%)	0.994			
Remote infection	1 (10%)	2 (2.9%)	0.341			

Bold text indicates a statistically significant difference with a *P*-value < 0.1 in univariate analysis and with a *P*-value < 0.05 in multivariate analysis

Continuous parameters are presented as mean (SD)*, and categorical parameters are presented as n (%), *P* < 0.05

## Discussion

In the present study, we examined POI in 213 cases of CRC surgery. The incidence of POI was 21 (9.9%). This frequency was almost the same as previously reported. Cases with POI showed a significant extension of hospital stay: 21.7 days compared to 14.7 days for non-POI cases. Previous reports have also reported that POI leads to extended hospital stays. Furthermore, in this study, a low SMI was an independent predictor of POI. To our knowledge, this is the first report on the relationship between POI and a low SMI. We report that SMI is a useful predictive marker for the occurrence of POI.

To date, several risk factors of POI have been reported, including hypoalbuminemia, lung disorders, male sex, a large amount of bleeding, and a long operation time [4, 8, 10–12, 23]. The implementation of standardized accelerated postoperative care pathways, namely oral antibiotic bowel preparation, early NG tube removal, early ambulation, early oral feeding, patient education, opioid-sparing epidural analgesia, perioperative fluid management, and minimally invasive surgical techniques has been reported to reduce the risk of POI [8, 24]. However, despite the widespread implementation of these measures, the incidence of POI for CRC is still reported to be 10%–30% after abdominal surgery [4, 8] and 15%–30% after colon and rectal surgery [10–12]. Therefore, predicting POI is very important in CRC surgery.

In the present study, univariate and multivariate analyses demonstrated that bleeding was associated with POI. However, the relationship between the POI and the previously reported non-bleeding data (hypoalbuminemia, lung disorders, male sex, and a long operation time) has not been elucidated in this study. Regarding the influence of bleeding on POI, it has been reported that an increase in hemoglobin drop due to the addition of colloidal fluid for intraoperative bleeding causes electrolyte imbalance, inducing intestinal edema [23]. Our results also demonstrated an association between bleeding and POI.

PSM was performed to further reduce the selection bias. As a result, a low SMI was found to be the only independent POI predictor in our examination of 78 matched cases.

The importance of sarcopenia in perioperative management has been shown in recent years, and there are a number of reports on the relationship between sarcopenia and postoperative complications. Lieffers et al. reported that 39% of 234 CRC surgery patients had sarcopenia, and the sarcopenia group showed a longer postoperative stay and more frequent infectious complications than the non-sarcopenia group [16]. Furthermore, the association between sarcopenia and postoperative complications has also been reported in patients with other cancer types such as esophageal cancer, gastric cancer and pancreatic cancer [25–28].

We had some results that appeared to differ from previous reports. First, the majority of the studies in literature reports a significant association between postoperative all complications and sarcopenia while it was not found in our study. Many previous reports have described an association between sarcopenia and remote infection, rather than with SSI including suture failure [17, 18, 29]. As well as these reports, the low SMI group had significantly more cases of severe pulmonary comorbidity preoperatively (p = 0.034), with a trend toward remote infection being more common in postoperative complications in our study. On the other hand, DM comorbidity preoperatively was significantly more frequent in the normal group (p = 0.007) and SSI and anastomotic leakage tended to be more common in the normal group. We considered it was for these reasons that there was no significant association between sarcopenia and all complications. Second, Vanera et al. reported an association between POI and anastomotic leakage while it was not found in our study. When anastomosis leakage occurs, inflammation spills over into the extensive abdominal cavity, which reduces intestinal peristalsis, which is thought to be associated with POI. In this study, 4 of the 17 cases with anastomosis leakage were Gr3b cases. As there were few cases of POI caused by diffuse peritonitis, such as Gr3b, it was considered that there was no association between POI and anastomotic leakage in this study.

However, to our knowledge, no report on the relationship between low SMI and POI has yet been published. There have been recent reports of decreased intestinal peristalsis in patients with sarcopenia. According to the report by Vaes et al., the generation of contractile forces by the gastrointestinal musculature is further regulated by the smooth muscle contractile activity, and the enteric nervous system, consisting of the myenteric plexuses and the interstitial cells of Cajal. In cancer patients with sarcopenia, a decreased expression of a contractile smooth muscle marker called smoothelin and the accumulation of collagen around the intestinal plexus are both known to occur, thus resulting in an impairment of both the intestinal smooth muscle contractile function and its regulation [30]. An in vivo study showed that the loss of smoothelin resulted in irregular slow wave patterns, impaired intestinal contraction, and hampered intestinal transit [31]. It was also reported that structural alterations in the myenteric plexus in tumor-bearing rats resulted in decreased upper gastrointestinal transit [32].

These previous findings therefore suggest that patients with sarcopenia already have factors that predispose them to intestinal motility disorders, so they may be prone to developing POI induced by surgical intervention.

Our present findings suggested that sarcopenia in cancer patients was associated with the development of POI. It is important to predict the occurrence of POI, and by promoting intestinal peristalsis and inserting an NG tube for postoperative nausea, aspiration due to ileus can be prevented. Preoperative sarcopenia patients are at high risk of developing POI, so it is important to take appropriate measures, such as promoting intestinal peristalsis early after surgery and inserting a NG tube early when symptoms appear, to prevent POI.

However, several limitations associated with the present study warrant mention. First, this study was retrospective and conducted in a single institution with a relatively small sample size. Second, the definition of a low SMI was determined by a BIA, and we did not investigate muscle power or function. Currently, there are no clear criteria for sarcopenia in Japanese patients, so it is also necessary to consider whether or not the SMI is appropriate for defining sarcopenia. Finally, although we determined cut-off values for the SMI based on the results of ROC curve analyses as an objective statistical method, an optimal standardized method for determining optimal cut-off values should be established. Further prospective studies with larger numbers of patients are needed to validate the utility of the preoperative SMI for predicting POI.

## Conclusion

A low SMI was an independent predictor of POI. The measurement of the preoperative SMI by the BIA method is very simple, and is considered a useful approach to predicting the onset of POI in patients after CRC surgery.

## Data Availability

The datasets used and/or analyzed during the current study are available from the corresponding author on reasonable request.
